# 2450. Clinical Outcomes of *Candida auris* Infections at an Academic Medical Center

**DOI:** 10.1093/ofid/ofad500.2068

**Published:** 2023-11-27

**Authors:** Yifan Wang, Emily N Drweiga, Larry H Danziger, Rodrigo M Burgos

**Affiliations:** University of Illinois at Chicago, College of Pharmacy, Chicago, Illinois; University of Illinois at Chicago College of Pharmacy, Chicago, Illinois; University of Illinois at Chicago, Chicago, IL; University of Illinois at Chicago, Chicago, IL

## Abstract

**Background:**

*C. auris (CA)* is a globally emerging fungus with a high case fatality rate (CFR). Cases of *CA* infection have been reported around US with a concentration in large metropolitan areas, but there is still little of data evaluating the prevalence and outcomes associated with *CA*. The purpose of this study was to evaluate the characteristics and outcomes in patients (pts) with *CA* isolation at a large urban academic medical center.

**Methods:**

This was a retrospective, observational cohort study of hospitalized pts with a positive culture with *CA* between Oct 2020 and Dec 2021 via VITEK MS. We included pts ≥18 years of age and excluded those with only a positive surveillance screen from axillary swab. Demographic and clinical data at the time of *CA* isolation and up to 30 days or hospital discharge were collected from the electronic medical record. The primary outcome was 30-day CFR in patients with a culture positive for *CA*.

**Results:**

We identified 37 patients, and 27 met inclusion. Of all included patients, 17 (63%) presented from acute or subacute long-term care facilities. 11 (40.7%) had *CA* isolated from a blood culture. 8/11 (72.7%) bloodstream infections were catheter-associated. Pts with fungemia had a median [IQR] length of antifungal treatment of 20 [6.5, 49.5] days, and all received micafungin. The median [IQR] days between index blood culture and first negative blood culture was 4 [3, 6.25]. 1 patient with fungemia never cleared repeat cultures despite treatment. Of all included pts, 7 of 27 (25.9%) expired within 30 days of *CA* isolation. The 30-day case fatality was 45.5% (5/11) in those with fungemia and 12.5% (2/16) in those with isolation from other sites.

**Conclusion:**

In our study, we report a similar institutional CFR of *CA* infections compared to the crude CFR within the U.S. We also report a higher CFR associated with *CA* fungemia compared to patients without positive blood cultures. These findings should be validated with a larger study population.

**Disclosures:**

**Larry H. Danziger, PharmD**, Ferring Pharmacetuicals: Advisor/Consultant **Rodrigo M. Burgos, PharmD, MPH**, Janssen Therapeutics & Vaccines: Grant/Research Support|Merck & Co., Inc.: Grant/Research Support|ViiV Healthcare: Advisor/Consultant

